# Development and validation of a deep learning model for detection of breast cancers in mammography from multi-institutional datasets

**DOI:** 10.1371/journal.pone.0265751

**Published:** 2022-03-24

**Authors:** Daiju Ueda, Akira Yamamoto, Naoyoshi Onoda, Tsutomu Takashima, Satoru Noda, Shinichiro Kashiwagi, Tamami Morisaki, Shinya Fukumoto, Masatsugu Shiba, Mina Morimura, Taro Shimono, Ken Kageyama, Hiroyuki Tatekawa, Kazuki Murai, Takashi Honjo, Akitoshi Shimazaki, Daijiro Kabata, Yukio Miki

**Affiliations:** 1 Department of Diagnostic and Interventional Radiology, Graduate School of Medicine, Osaka City University, Osaka, Japan; 2 Department of Breast and Endocrine Surgery, Graduate School of Medicine, Osaka City University, Osaka, Japan; 3 Department of Premier Preventive Medicine, Graduate School of Medicine, Osaka City University, Osaka, Japan; 4 Department of Gastroenterology, Graduate School of Medicine, Osaka City University, Osaka, Japan; 5 Department of General Practice, Osaka City University Hospital, Osaka, Japan; 6 Department of Medical Statistics, Graduate School of Medicine, Osaka City University, Osaka, Japan; Medical University of Vienna, AUSTRIA

## Abstract

**Objectives:**

The objective of this study was to develop and validate a state-of-the-art, deep learning (DL)-based model for detecting breast cancers on mammography.

**Methods:**

Mammograms in a hospital development dataset, a hospital test dataset, and a clinic test dataset were retrospectively collected from January 2006 through December 2017 in Osaka City University Hospital and Medcity21 Clinic. The hospital development dataset and a publicly available digital database for screening mammography (DDSM) dataset were used to train and to validate the RetinaNet, one type of DL-based model, with five-fold cross-validation. The model’s sensitivity and mean false positive indications per image (mFPI) and partial area under the curve (AUC) with 1.0 mFPI for both test datasets were externally assessed with the test datasets.

**Results:**

The hospital development dataset, hospital test dataset, clinic test dataset, and DDSM development dataset included a total of 3179 images (1448 malignant images), 491 images (225 malignant images), 2821 images (37 malignant images), and 1457 malignant images, respectively. The proposed model detected all cancers with a 0.45–0.47 mFPI and had partial AUCs of 0.93 in both test datasets.

**Conclusions:**

The DL-based model developed for this study was able to detect all breast cancers with a very low mFPI. Our DL-based model achieved the highest performance to date, which might lead to improved diagnosis for breast cancer.

## Introduction

Among all types of cancer, breast cancer has both the highest incidence (24%) and highest mortality (15%) in women around the world [1]. Mammography uses low-energy X-rays to identify abnormalities in the breast. For women who are at average risk for breast cancer, most of the benefit of mammography results from biennial screening during ages 50 to 74 years [2]. Of all age groups, women aged 60 to 69 years are most likely to avoid death from breast cancer through mammography screening [2]. The sensitivity and specificity of mammography screening for breast cancer are reported to be 77–78% and 89–97%, respectively [3,4]. Although breast cancer screening with mammography is considered effective in reducing breast cancer-related mortality, interpreting mammograms is a delicate task and prone to errors, with at least 25% of detectable cancers being missed [5–9]. Detecting subtle regions such as microcalcifications and focal asymmetric density (FAD) in particular pose difficult hurdles for physicians. Several computer-aided detection (CAD) systems have been developed to overcome this problem and provide physician support. Initially, studies showed that a single-reading with CAD systems could be an alternative to double-reading [10–13]. However, studies have since concluded that the cost-effectiveness of screenings had not improved, mainly because of the low specificity of traditional CAD systems [4,14,15].

Recently, the application of convolutional neural networks, one field of deep learning (DL), has led to dramatic improvements in visual object recognition, detection, and segmentation [16,17]. In this study, we adopted to create a detection-based DL model that could detect all the findings that breast cancer can present, including not only masses, but also architectural distortion and microcalcifications. While masses can be segmented, other findings are difficult to segment because it is difficult to accurately delineate the boundary between normal and abnormal areas. Therefore, we thought that a bounding box detection AI model was the most suitable for our study. Models using DL have routinely surpassed the performance of traditional methods due to their automated feature extraction [18]. These dramatic improvements have caught the eye of researchers in several fields, including mammography [19–37]. In addition to those that detect breast cancer [19–32], there are studies to predict the risk of breast cancer from mammography [33–35]. For patients with breast cancer, there are models which estimate the expression of receptors involved in chemotherapy selection [36], and those that predict pathological types [37]. Sensitivity for studies detecting breast cancer was found to be in the range of 0.76–0.97, with a mean number of false positive indications per image (mFPI) of 0.48–3.56. Sensitivity and mFPI are often used to evaluate the detection model, where the mFPI is the average number of false positive lesions displayed by the model for a single image. There is a trade-off between sensitivity and mFPI, since the greater the number of false positive lesions presented by the model, the higher the sensitivity. For this reason, a higher sensitivity with a lower mFPI is desirable in a model intended to help physicians interpret mammograms for the benefit of their patients. The purpose of the present study was to train and validate a state-of-the-art DL-based model to detect breast cancer with higher performance than existing models.

## Methods

### Study design

First, a DL-based model for detecting breast cancer on mammograms was trained and validated using retrospectively collected mammograms annotated by the radiologists with the locations of malignant lesions. Second, the model was tested with independent datasets for the detection of breast cancers. The Ethical Committee of Osaka City University Graduate School of Medicine comprehensively reviewed and approved the protocol of this study. Since the mammograms had been acquired during daily clinical practice, the need for informed consent was waived by the ethics board. We have created this article in compliance with the Transparent Reporting of a multivariable prediction model for Individual Prognosis Or Diagnosis (TRIPOD) statement [38].

There are two possible ways to label mammograms when developing an AI model for breast cancer screening. The mammograms can be labelled using BI-RADS grading or pathology [39]. The advantage of the former is that a large dataset of mammograms can be prepared since pathology results are not required, but on the other hand, BI-RADS grading is known to be more subjective than the pathology result [40]. In other words, if we created an AI model with BI-RADS as a label, the AI model may output false positives for mammograms that have a high grading in BI-RADS but are not pathologically breast cancer.

### Datasets

To train, validate, and test the DL-based model, four datasets were used: a hospital development dataset, a hospital test dataset, a clinic test dataset, and the Digital Database for Screening Mammography (DDSM) dataset [41–43]. Mammograms for the hospital development dataset and the hospital test dataset were retrospectively collected from patients who were surgically diagnosed with breast cancer at Osaka City University Hospital, which provides secondary care. Mammograms in the clinic test dataset were collected from patients who underwent mammography screening at Medcity21 Clinic, a provider of preventive medicine. The hospital development dataset and hospital test dataset were collected consecutively from January 2006 through December 2016 and from January 2017 through December 2017, respectively. The clinic test dataset was collected consecutively from April 2014 through March 2017.

Malignant mammograms were collected from both sides of patients with bilateral breast cancer and the affected side of patients with unilateral breast cancer for the hospital test, hospital development, and clinic test datasets. Nonmalignant mammograms for the hospital development and hospital test datasets were collected from the healthy side of patients with unilateral breast cancer. The mammograms were diagnosed as nonmalignant in preoperative screening by five surgeons who specialized in breast surgery. Nonmalignant mammograms in the clinic test dataset were collected from both sides of healthy patients, and the healthy side of patients who had pathologically diagnosed unilateral breast cancer. Nonmalignancy was then confirmed with 2 years of follow-up mammograms by two radiologists who had 18 years and 10 years of experience interpreting mammography.

Since the study included breast cancer patients who visited each institution for the first time, none of the datasets had overlaps. Both left and right mediolateral oblique (MLO) and craniocaudal (CC) images were collected, if available.

### Ground truth labelling

Malignant lesions on the affected side of mammograms in the hospital development dataset were annotated by two radiologists who had 6 years and 5 years of experience interpreting mammography. Mammograms were annotated with bounding boxes and labelled as mass, calcification, distortion, and FAD with reference to ultrasound, radiological, biopsy, and surgical reports. When there was disagreement between the radiologists, consensus was achieved by discussion. In addition, they could consult with a third expert if needed. Mammograms with no findings in the affected side were excluded. The density of the mammary glands on all mammograms was assessed by the same radiologists according to the BI-RADS [39] in consensus. This assessment was performed on a mammogram basis, rather than a patient basis. All malignant findings (mass, calcifications, FAD, and architectural distortion) of each cancer were merged into one bounding box. Mammograms with multiple breast cancers would have multiple bounding boxes.

Malignant lesions on the affected side of mammograms in the hospital test dataset and the clinic test dataset were annotated in the same manner as the hospital development dataset by two radiologists who had 6 years and 12 years of experience interpreting mammography.

Ground truth labelling for the publicly available DDSM development dataset was as follows. The Curated Breast Imaging Subset of the DDSM (CBIS-DDSM) [41–43] is an updated and standardized version of the DDSM. In this dataset, all mammograms include pathologically verified breast cancer; a segmentation of malignant findings is included. Malignant mammograms were collected from both sides of patients with bilateral breast cancer and the affected side of patients with unilateral breast cancer from the CBIS-DDSM. Bounding boxes were created from the longest diameter in the vertical and horizontal directions of the malignant segmentation. All malignant findings (mass, calcifications, FAD, and architectural distortion) of each cancer on the same mammogram were merged into one bounding box. Mammograms with multiple breast cancers would have multiple bounding boxes.

### Training and validation of the model

**Fig 1:**
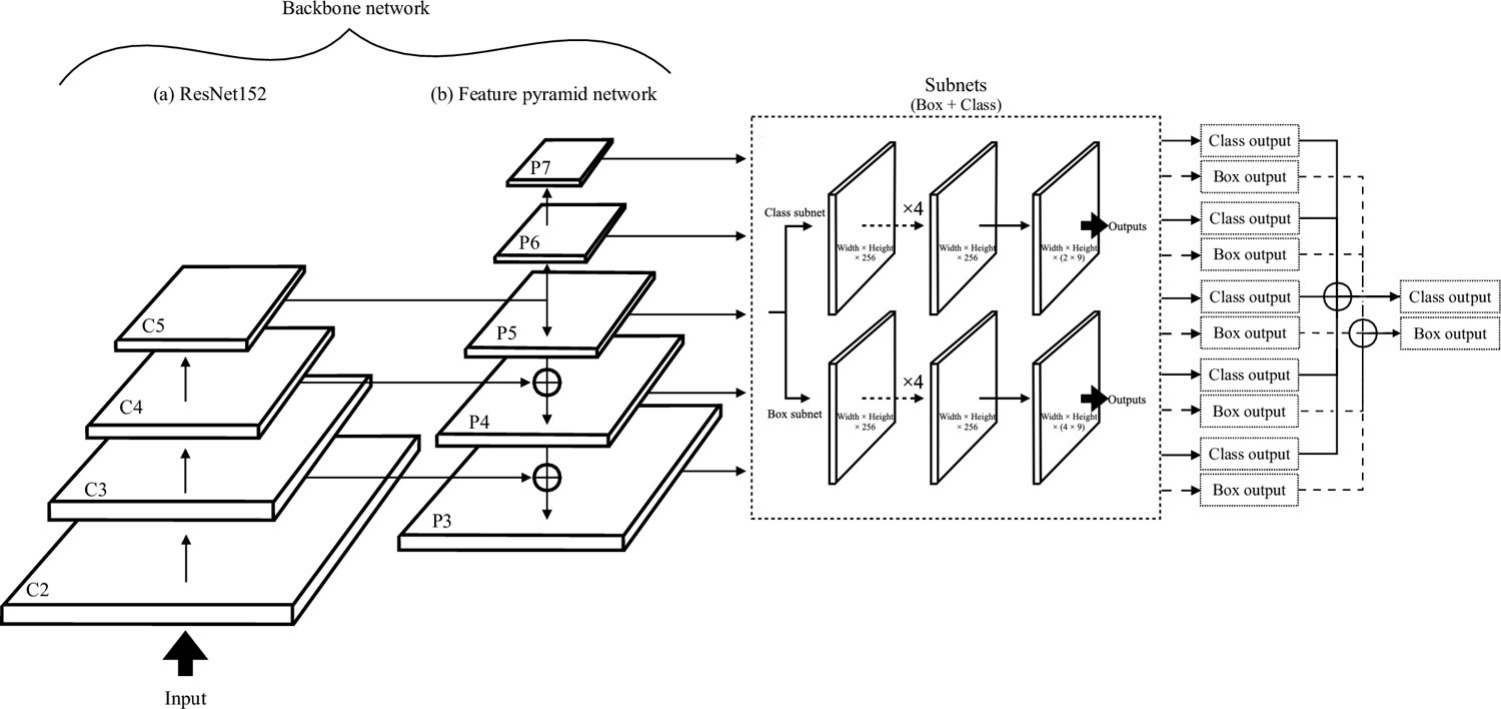
Structure of the RetinaNet in our study. This is the overview of the model in this research [44]. The backbone network was composed of (a) ResNet152 [45] and (b) the Feature Pyramid Network (FPN) [46]. The ResNet and FPN have a bottom-up (downsampling) pathway and a top-down (upsampling) pathway, respectively. The sizes of the processing image in ResNet have 4 levels (C2, C3, C4, C5) and FPN is 5 levels (P3, P4, P5, P6, P7) with 256 channels. Both ResNet and FPN were connected with lateral connections. C3 connects to the P4-P3 pathway and C4 connects to the P5-P4 pathway. Nine translation-invariant anchors, each of a different size, are used at each level of FPN. Each anchor is assigned a 2-class length of one-hot vector and a 4-dimensional vector of box regression targets. The class subnet is used for classifying anchor boxes. It estimates the probability of object presence at each spatial position for the 9 anchors and 2 object classes (malignant or nonmalignant). The class subnet is a small fully convolutional network attached to each level of the FPN. The subnet applies four 3 × 3 convolution layers with 256 channels each, and an additional 3 × 3 convolution layer with 2 × 9 filters to feature maps from each level of FPN. Finally, sigmoid activations are attached to output the 2 × 9 predictions. The box subnet is also attached to each level of FPN. The box subnet is identical to the classification subnet except that it terminates in 4 × 9 linear outputs per spatial location. The box subnet is used for regressing the existing offset between a nearby ground-truth box and the anchor box.

A DL-based model was developed using RetinaNet [44] to detect lesions and evaluate the probability of breast cancer in mammograms. RetinaNet is a regression-based, unified framework with a backbone and two subnetworks which detect and classify objects. The backbone network used in our study was ResNet152 [45] with a feature pyramid network [46]. The ResNet has four downsampling levels and the FPN has five upsampling levels, each with 256 channels. The backbone network computes convolutional feature maps of an entire input mammogram. The first subnetwork, called “class subnet,” classifies the output of the backbone network as either malignant or not malignant. The second subnetwork, called “box subnet,” performs convolutional bounding box regression. This network adopted focal loss for class subnet and L1 loss for box subnet. Focal loss focuses training on a sparse set of hard examples and prevents the vast number of easy negatives from overwhelming the detector during training. RetinaNet is tuned to classify sites outside the adenoma bounding box as background. For example, mammary glands in a different location from the breast cancer on mammograms will be treated as a true negative. Through these processes, the model extracts features that are unique to breast cancer. For structural details, see Fig 1; the source code is available online [47]. This model was built in the TensorFlow framework [48].

The RetinaNet-based model was trained and validated with both malignant and nonmalignant mammograms from the hospital and DDSM development datasets. The images and bounding boxes for the training and validation of the RetinaNet were prepared as follows: (i) Mammograms were downscaled to 800 pixels on the longest side while maintaining the aspect ratio. This pixel size was the minimum value of the longest side of the mammograms in the development datasets, so we downsized larger images in order to be able to include as many images as possible. (ii) The shorter side of the mammograms was padded black to 800 pixels. (iii) Bounding boxes were also resized to match each downscaled malignant mammogram.

The mammograms and bounding boxes in the two development datasets were divided into training and validation with five-fold cross-validation. The RetinaNet was trained for 100 epochs, and the learning parameters when the value of the validation-loss function was the lowest was adopted. The learning progress of the DL-based model was monitored by both the value of the validation-loss function and the sensitivity of detection for breast cancers when the intersection over union (IoU) was set to 0.5. As optimizers, SGD and Adam were evaluated with their default parameters. All images were augmented using random rotation from –0.1 radians to 0.1 radians, with a random shift of 10% (80 pixels), a random shear of 10% (80 pixels), and random scaling from –10% (–80 pixels) to 10% (80 pixels), then flipped vertically and horizontally.

The model was programmed to display bounding boxes on the area of suspected cancer in a mammogram, along with a malignancy likelihood ratio from 0 to 1. The model can adjust the number of boxes that are presented as well as the cut-off of the malignancy likelihood ratio of the proposed boxes. (S1 Fig in S6 File) We have trained other AI models as well. Descriptions of these models are available in the supplementary materials in S6 File.

### Model performance test

A lesion-based performance test was performed on the hospital and clinic test datasets. The test was performed as follows: (1) All mammograms were prepared as described for the training and validation of the model, steps (i) to (iii). (2) The trained DL-based model with the lowest validation-loss value was applied to these processed mammograms. (3) The overlap of the bounding box presented by the model and the radiologist annotated ground truth was calculated; this is known as the IoU. When the IoU was 0.3 or higher, the model had correctly identified the known malignancy. This IoU was chosen based on the results of a previous study [28]. Until every ground truth was detected, the model continued to present the boxes from highest model-estimated malignancy to lowest, lowering the threshold of malignancy for presented boxes. These boxes and the malignancy likelihood ratios presented by the model were used to evaluate the detection performance.

Additionally, an image-based performance test was performed on the hospital and clinic test datasets to assess the model’s ability to discriminate between malignancy and nonmalignancy. The DL-based model’s threshold of malignancy was determined by the Youden Index for this evaluation. The test was performed as follows: (1) All mammograms were prepared as described for the training and validation of the model, steps (i) to (iii). (2) The model was applied to these processed mammograms in the test datasets. (3) A malignant mammogram with annotations with an IoU greater than or equal to 0.3 for a ground-truth lesion was defined as a true positive image, a malignant mammogram with annotations with an IoU less than 0.3 for a ground-truth lesion was defined as a false negative image, a nonmalignant mammogram with no annotations on a mammogram was defined as a true negative image, and a nonmalignant mammogram with one or more annotations was defined as a false positive image.

### Statistical analysis

In the lesion-based performance test, we evaluated whether the bounding boxes proposed by the model accurately identified malignant lesions in mammograms using the free-response receiver operating characteristic (FROC) [49] curves. In the FROC, the vertical axis shows sensitivity; the horizontal axis shows mFPI. Thus, the FROC curve shows sensitivity as a function of the number of false positive lesions. Sensitivity was defined as the number of true positive lesions that the model presented divided by the number of all true positive lesions. The mFPI was defined as the number of false positive lesions that the model presented divided by the number of all mammograms in the dataset. Additionally, in the image-based performance test, we evaluated the model using the partial area under the curve (AUC), accuracy, sensitivity, specificity, positive predictive value (PPV), and negative predictive value (NPV).

Two of the authors (D.U. and D.K.) performed all analyses using R, version 3.6.0. The FROC curves were plotted by R. All statistical inferences were performed with a two-sided 5% significance level.

#### Patient and public involvement

There was no direct patient or public involvement in this study.

## Results

### Datasets

The hospital development dataset included 3179 images (897 patients; age range, 25–97 years; mean age ± standard deviation, 58 ± 12 years) after excluding 367 images (170 MLO and 197 CC images) with no malignant findings. There were 1448 malignant and 1731 nonmalignant images. There were 1412 digital and 1767 scanned film images. Regarding breast density, 472 images were almost entirely in fat, 993 in scattered fibroglandular tissue, 999 in heterogeneously dense tissue, and 715 in extremely dense tissue. The malignant findings were as follows: 812 masses, 703 calcifications, 389 FAD, and 520 architectural distortions.

The publicly available DDSM development dataset included a total of 1457 malignant images each with one bounding box. All images were collected from the CBIS-DDSM.

In total, 4636 mammograms (2905 malignant and 1731 nonmalignant images) from the hospital and DDSM development datasets were used to develop the model.

The hospital test dataset included a total of 491 images (139 patients; age range, 33–92 years; mean age ± standard deviation, 59 ± 13 years) after excluding 49 images (22 MLO and 27 CC images) without malignant findings on the affected mammograms. In total, there were 225 malignant and 266 nonmalignant images. Among these 491 images, there were 327 digital and 164 scanned film images. Regarding breast density, 74 images were almost entirely in fat, 180 in scattered fibroglandular tissue, 161 in heterogeneously dense tissue, and 76 in extremely dense tissue. In total, 230 breast cancers were detected in 225 malignant images (two malignant cancers were detected in five patients). The malignant findings were as follows: 103 masses, 83 calcifications, 74 FAD, and 93 architectural distortions.

The clinic test dataset included a total of 2821 images (865 patients; age range, 32–84 years; mean age, 52 ± 8 years) after excluding 1358 images with no follow-up and one CC image with no malignant findings. There were 37 malignant and 2784 nonmalignant images. All images were digital. Regarding breast density, 435 images were almost entirely in fat, 962 in scattered fibroglandular tissue, 983 in heterogeneously dense tissue, and 441 in extremely dense tissue. No mammograms showed multiple cancers. The malignant findings were as follows: six masses, 19 calcifications, 11 FAD, and six architectural distortions.

**Fig 2:**
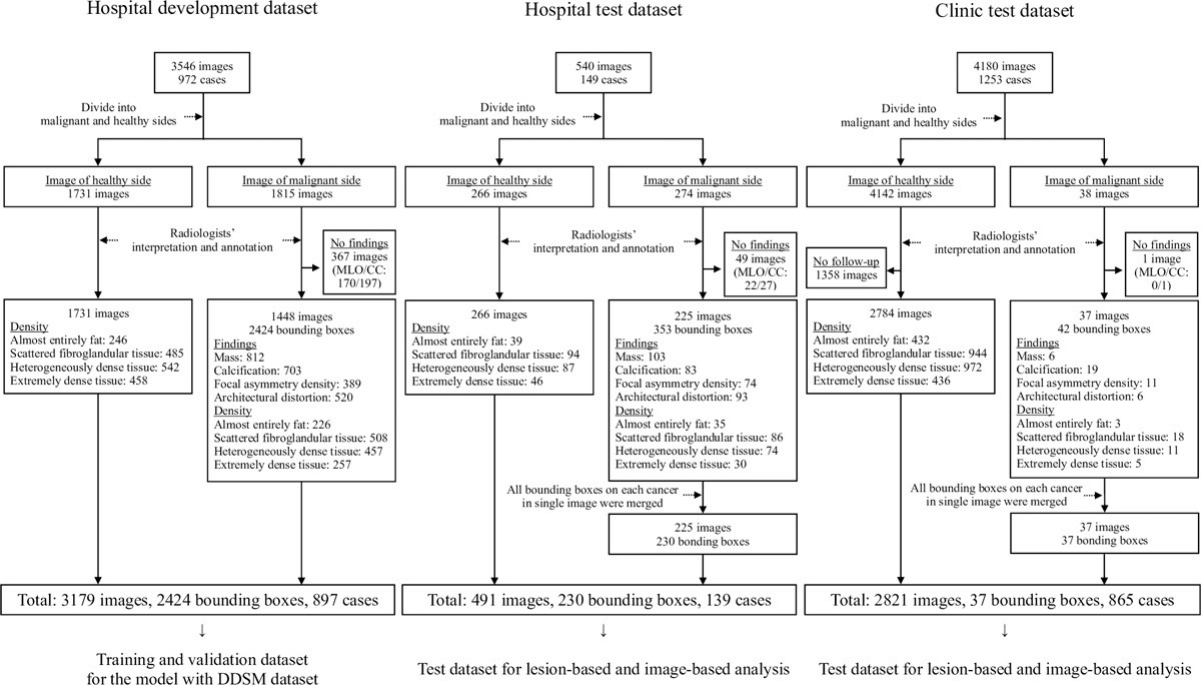
Flowcharts of the eligibility criteria. DDSM: Digital database for screening mammography; MLO: Mediolateral oblique; CC: Craniocaudal.

A flowchart of the eligibility criteria of the hospital and clinic datasets is shown in Fig 2. Detailed demographic information of the development and test datasets is provided in Tables 1 and 2, respectively.

**Table 1:** Characteristics of the development datasets.

Characteristics	Hospital development dataset	DDSM development dataset
Patient information		
No. of patients	897	752
No. of female	897	752
Mean age ± standard deviation (y)	58 ± 12	NA
No. of mammograms	3179	1457
No. of malignant mammograms	1448	1457
No. of nonmalignant mammograms	1731	0
No. of MLO images	1706	681
No. of CC images	1473	776
No. of digital images	1412	0
No. of scanned film images	1767	1457
No. of malignant findings		
Mass	812	784
Calcification	703	673
Focal asymmetry density	389	0
Architectural distortion	520	320
Background mammary glands density		
Almost entirely fat	472	204
Scattered fibroglandular tissue	993	569
Heterogeneously dense tissue	999	461
Extremely dense tissue	715	223

MLO: Mediolateral oblique.

CC: Craniocaudal.

**Table 2:** Characteristics of the test datasets.

Characteristics	Hospital test dataset	Clinic test dataset
Patient information		
No. of patients	139	865
No. of female	139	865
Mean age ± standard deviation (y)	59 ± 13	52 ± 8
No. of mammograms	491	2821
No. of malignant mammograms	225	37
No. of nonmalignant mammograms	266	2784
No. of digital images	327	2821
No. of scanned film images	164	0
No. of MLO images	256	1475
No. of CC images	235	1346
Background mammary glands density		
Almost entirely fat	74	435
Scattered fibroglandular tissue	180	962
Heterogeneously dense tissue	161	983
Extremely dense tissue	76	441
Cancer information		
No. of cancers in all mammograms	230	37
Size		
Carcinoma in situ	17	3
1–10 mm	37	6
11–20 mm	82	20
21–50 mm	86	8
>50 mm	8	0
No. of malignant findings		
Mass	103	6
Calcification	83	19
Focal asymmetry density	74	11
Architectural distortion	93	6
Pathology		
Invasive ductal carcinoma	179	30
Ductal carcinoma in situ	17	3
Invasive lobular carcinoma	19	4
Mucinous carcinoma	4	0
Apocrine carcinoma	2	0
Encapsulated papillary carcinoma	2	0
Squamous cell carcinoma	2	0

MLO: Mediolateral oblique.

CC: Craniocaudal.

### Model development

The DL-based model was trained and validated on the two development datasets with five-fold cross-validation. The highest performance was observed when the optimizer used was Adam. The validation-loss function minima was obtained at 52 epochs.

### Model performance test

**Fig 3:**
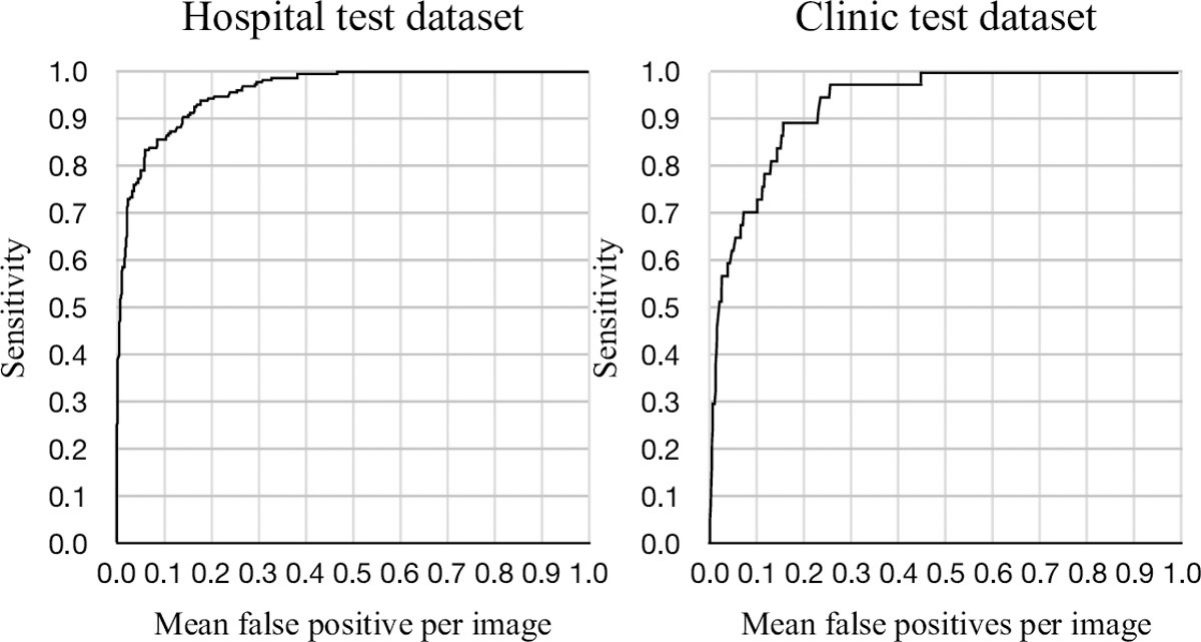
Free-response receiver operating characteristic curves for the hospital test dataset and clinic test dataset. These free-response receiver operating characteristic curves show a lesion-based analysis. The vertical axis shows the sensitivity of correctly detected breast cancer lesions by the model. The horizontal axis shows the mean number of false-positive lesions per mammogram. The partial area under the curve with 1.0 mean false positive indications per image was 0.93 (0.90–0.95) in the hospital dataset and 0.93 (0.90–0.96) in the clinic test dataset.

**Fig 4:**
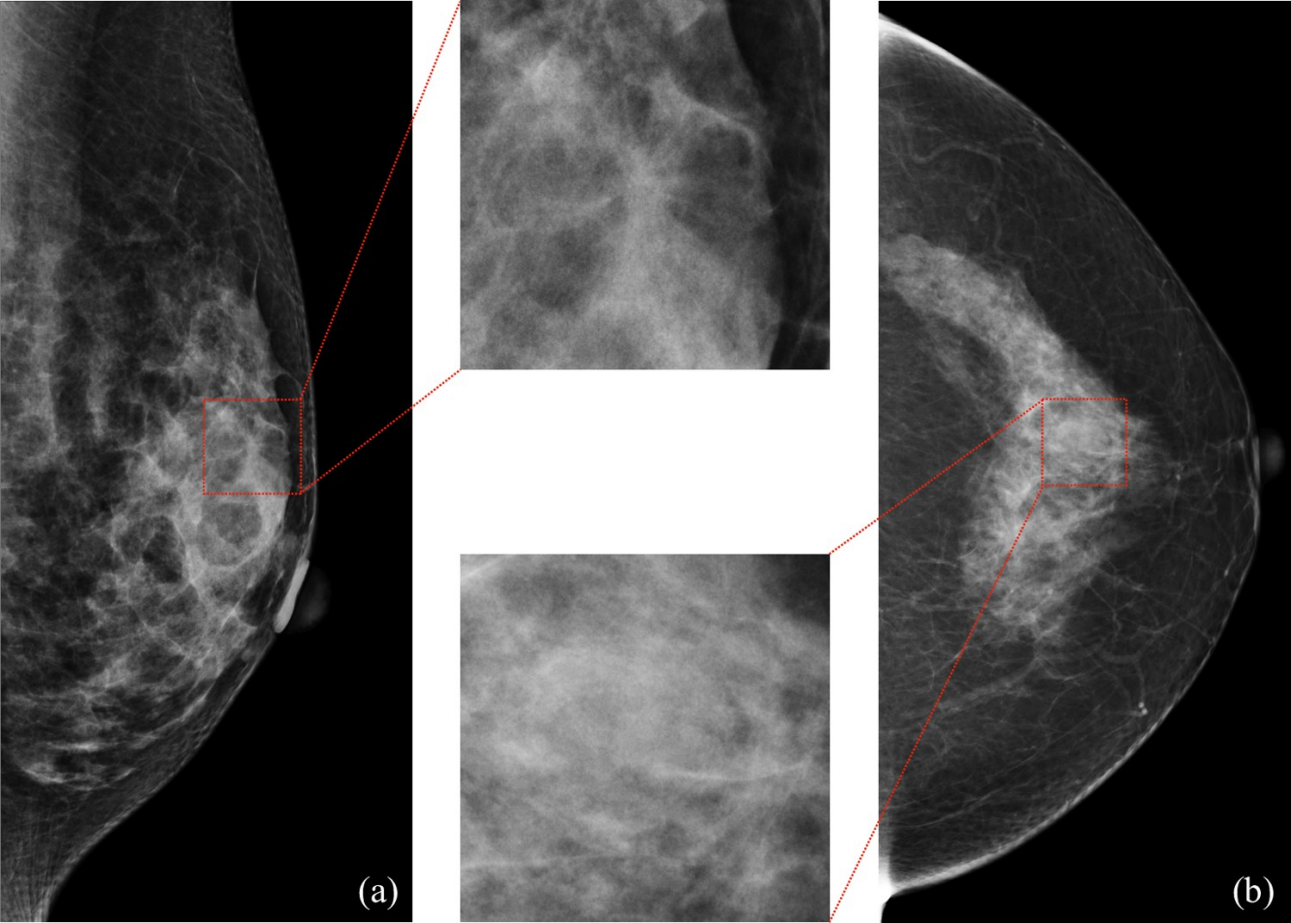
The most difficult cancers for the model to detect. (a) A 22-mm (long-axis diameter) cancer (box) presented architectural distortion with heterogeneously dense tissue in the mammary glands of a 41-year-old woman. The malignancy likelihood ratio was 0.24. (b) A 11-mm (long-axis diameter) cancer (box) presented a mass with scattered fibroglandular tissue in the mammary glands of a 58-year-old woman. The malignancy likelihood ratio was 0.33.

The lesion-based performance of the DL-based model had a sensitivity of 1.00 with 0.47 mFPI in the hospital test dataset, and 1.00 with 0.45 mFPI in the clinic test dataset (Fig 3). The partial AUC with an mFPI of 1.0 was 0.93 (0.90–0.95) in the hospital dataset and 0.93 (0.90–0.96) in the clinic test dataset. Every malignancy detected was the lesion with the highest likelihood ratio in the mammogram. In cases in which there were two malignant findings in one mammogram, both lesions detected were the ones with the highest and second highest probability of malignancy. The most difficult cancers for the model to detect in the hospital and clinic test datasets are shown in Fig 4. Although these lesions had the highest probability of malignancy in the mammograms, the malignancy likelihood ratios were the lowest of all true positive lesions (0.24 in the hospital test dataset and 0.33 in the clinic test dataset). Results applying other AI models are available in the supplementary materials in S6 File.

The image-based performance showed that the accuracy, sensitivity, specificity, PPV, and NPV were 0.86 (0.83–0.89), 0.84 (0.79–0.89), 0.88 (0.83–0.91), 0.85 (0.80–0.90), and 0.87 (0.82–0.90), respectively, in the hospital test dataset, and 0.85 (0.84–0.87), 0.84 (0.68–0.94), 0.85 (0.84–0.87), 0.07 (0.05–0.10), and 1.00 (0.99–1.00), respectively, in the clinic test dataset (Table 3).

**Table 3:** Results of the image-based performance of the model.

Characteristics	Hospital test dataset	Clinic test dataset
Accuracy	0.86 (0.83–0.89)	0.85 (0.84–0.87)
Sensitivity for diagnosis	0.84 (0.79–0.89)	0.84 (0.68–0.94)
Specificity for diagnosis	0.88 (0.83–0.91)	0.85 (0.84–0.87)
Positive predictive value	0.85 (0.80–0.90)	0.07 (0.05–0.10)
Negative predictive value	0.87 (0.82–0.90)	1.00 (0.99–1.00)
Sensitivities by mammary gland density		
Almost entirely fat	0.97 (0.85–1.00)	0.67 (0.09–0.99)
Scattered fibroglandular tissue	0.90 (0.81–0.95)	0.83 (0.59–0.96)
Heterogeneously dense tissue	0.77 (0.66–0.86)	0.91 (0.59–1.00)
Extremely dense tissue	0.70 (0.50–0.85)	0.80 (0.28–0.99)
Specificities by mammary gland density		
Almost entirely fat	0.87 (0.73–0.96)	0.84 (0.80–0.88)
Scattered fibroglandular tissue	0.81 (0.71–0.88)	0.79 (0.77–0.82)
Heterogeneously dense tissue	0.90 (0.81–0.95)	0.87 (0.85–0.89)
Extremely dense tissue	0.98 (0.88–1.00)	0.95 (0.93–0.97)

Note—Numbers in parentheses are 95% confidence intervals.

## Discussion

The results of the present study indicated that the proposed DL-based model could accurately detect all breast cancers on mammograms with 0.47 mFPI in the hospital test dataset and 0.45 mFPI in the clinic test dataset. To our knowledge, the model developed in this research represents state-of-the-art performance for detecting breast cancer.

In examining relevant prior research, we found fourteen studies [19–32] proposing DL-based models designed for detecting breast cancers on mammograms (not only for classifying lesions as malignant or nonmalignant). Specifically, McKinney *et al*. [29] achieved a multi-localization receiver operating characteristic of the partial AUC of 0.048 with a false positive rate of 10%. Even though they also used both normal and malignant images to train their model, our model has a lower mFPI and detects and classifies lesions at the same time rather than separately. Two studies [27,30] had performance comparable to our model. The reported lesion-based sensitivity in these studies was 0.76–0.97, with an mFPI of 0.48–3.56. Ribli *et al*. [30] achieved a sensitivity of 0.9 with a 0.3 mFPI for detecting breast cancer, while Jung *et al*. [27] achieved a sensitivity of 0.86–1.00 with a 0.5–3.0 mFPI for detecting only mass lesions of breast cancer. Our model achieved a higher sensitivity and a lower mFPI than have been reported previously. Although it is difficult to compare the model performance because of the differences in the test datasets, possible explanations for the performance of our model are the size and composition of the development dataset and the DL architecture. Our model was developed with 4636 mammograms (2905 malignant and 1731 nonmalignant images), while Ribli *et al*. [30] (2843 mammograms) and Jung *et al*. [27] (116–632 mammograms) developed their models using only malignant mammograms. It is possible that development with a larger number, as well as both malignant and nonmalignant images, resulted in a lower mFPI due to our model learning more about normal features [22]. With respect to the DL architecture, our model was developed using RetinaNet based on ResNet-152. RetinaNet is particularly useful when images for each of the classes (here malignant and nonmalignant) are likely to present in uneven numbers. Additionally, the variety of mammograms used to develop the model likely prevented overfitting. Overfitting is a result of learning that corresponds too closely to a particular development dataset and may therefore fail to fit additional data. In the present study, two datasets from different institutions were used, as were both converted-film and digital images.

With regard to the image-based performance of our DL-based model, it was relatively difficult for our DL-based model to detect malignant findings in denser breast tissues and calcifications. Similar results have been reported in other studies [21,31]. This is reasonable because the development datasets were annotated by radiologists, then the DL-based model extracted and learned features from these datasets. In other words, the performance of the model depends on the quality and quantity of the developing datasets. Another hypothesis for these difficulties is that malignant findings in denser mammary glands and calcifications are so subtle that they might have been lost when the mammograms were resized during the development process. Decreasing the compression ratio when developing model is worth investigating in the future.

Since our trained model is open source [47], it is possible to efficiently re-train a part of the trained model with new mammograms which are closer to the cohort of intended use [48]. Different countries and institutions have different cohorts of mammograms which may differ from those used to train the model for this study. Others may achieve better use of our trained model by fine-tuning it to fit their own purposes.

The study described here is not without limitations. We found that the clinic test dataset was largely dominated by normal cases, but still not as many as the real screening cohort. The number of false positives may be higher in the real screening cohort and its impact should be considered.

We developed and tested a model for the automated detection of breast cancer from mammograms using DL with RetinaNet. Our model was able to detect all breast cancers in the test datasets, regardless of type or tissue density, with a comparatively small mFPI. The trained model is open source and can be used worldwide. Our model is available free of charge with Apache License 2.0 [47].

## Supporting information

S1 File Data availability statement. (DOCX)

S2 File Image_Based-ClinicTest. (CSV)

S3 File Image_Based-HospTest. (CSV)

S4 File Lesion_Based-ClinicTest. (CSV)

S5 File Lesion_Based-HospTest. (CSV)

S6 File Supplemental_Materials_PLOSrev1_clear. (DOCX)
