# Supplementary material for: Development and validation of a deep learning model for detection of breast cancers in mammography from multi-institutional datasets
Source: PLoS One. 2022 Mar 24;17(3):e0265751. doi: 10.1371/journal.pone.0265751 (PMC8947392; doi:10.1371/journal.pone.0265751)
Supplement: S1 File — (DOCX) [file pone.0265751.s001.docx]

The code used to train and test the model is available at <https://github.com/detection-mammography>. Other individual participant data that underlie the results reported in this article (text, tables, and figures) are available in the Supporting Documents. The Digital database for screening mammography (DDSM) dataset is available at doi:10.1038/sdata.2017.177. Source mammography and pathology data are restricted because they include sensitive patient information. They can be made available with a methodologically sound proposal and only for analyses to achieve the aims in the approved proposal through the permission of Ethical Committee of Osaka City University Graduate School of Medicine (<http://www.med.osaka-cu.ac.jp/ocucrb>). For further details please contact [ethics@med.osaka-cu.ac.jp](mailto:ethics@med.osaka-cu.ac.jp).
