# Supplementary material for: Development and validation of a deep learning model for detection of breast cancers in mammography from multi-institutional datasets
Source: PLoS One. 2022 Mar 24;17(3):e0265751. doi: 10.1371/journal.pone.0265751 (PMC8947392; doi:10.1371/journal.pone.0265751)
Supplement: S6 File — (DOCX) [file pone.0265751.s006.docx]

**Title**

Development and validation of a deep learning model for detection of breast cancers in mammography from multi-institutional datasets.

**Supplemental Materials**

**Supplemental Methods**

We implemented two other AI models to detect breast cancer in mammography (YOLOv3-608 [1] and Faster-RCNN [2] with ResNet152). We evaluated whether the bounding boxes proposed by the models accurately identified malignant lesions in mammograms using the free-response receiver operating characteristic (FROC) curves [3] which are shown in supplemental Figure 2. The performances among the AI models were compared with the Delong test. The results are in supplemental Table 1.

In this study, we determined the most suitable hyperparameters for our final model’s optimizer, learning rate, image size, and batch size through the following steps. For the optimizer, we evaluated the stochastic gradient descent, Adam, and Adagrad; for the learning rate, the searching range for stochastic gradient descent was 0.001–0.05 and default parameters were applied for the other optimizers; for the image size, the searching range was 300, 500, 800, and 1000 pixels; for batch size, the searching range was 16–256.

Upon completion of the hyperparameter tuning, the final hyperparameters chosen were: optimizer = Adam, image size = 800 pixels, and batch size = 32

**Supplemental Tables**

**Supplemental Table 1: Model results using alternative architectures**

|  |  |  | 95% confidence interval | |  |
| --- | --- | --- | --- | --- | --- |
|  | Model | Partial AUC | Lower | Upper | P values |
| Hospital test dataset | RetinaNet | 0.93 | 0.91 | 0.95 | ref |
|  | YOLOv3 | 0.90 | 0.87 | 0.92 | <0.001 |
|  | Faster-RCNN | 0.89 | 0.86 | 0.92 | <0.001 |
| Clinic test dataset | RetinaNet | 0.93 | 0.90 | 0.96 | ref |
|  | YOLOv3 | 0.89 | 0.84 | 0.93 | 0.001 |
|  | Faster-RCNN | 0.88 | 0.83 | 0.94 | <0.001 |

**Supplemental Figures**

**Supplemental Figure 1 Examples of mammograms with bounding boxes proposed by our model.**

The numbers above the boxes are the malignant likelihood ratios. MLO: mediolateral oblique; CC: craniocaudal.


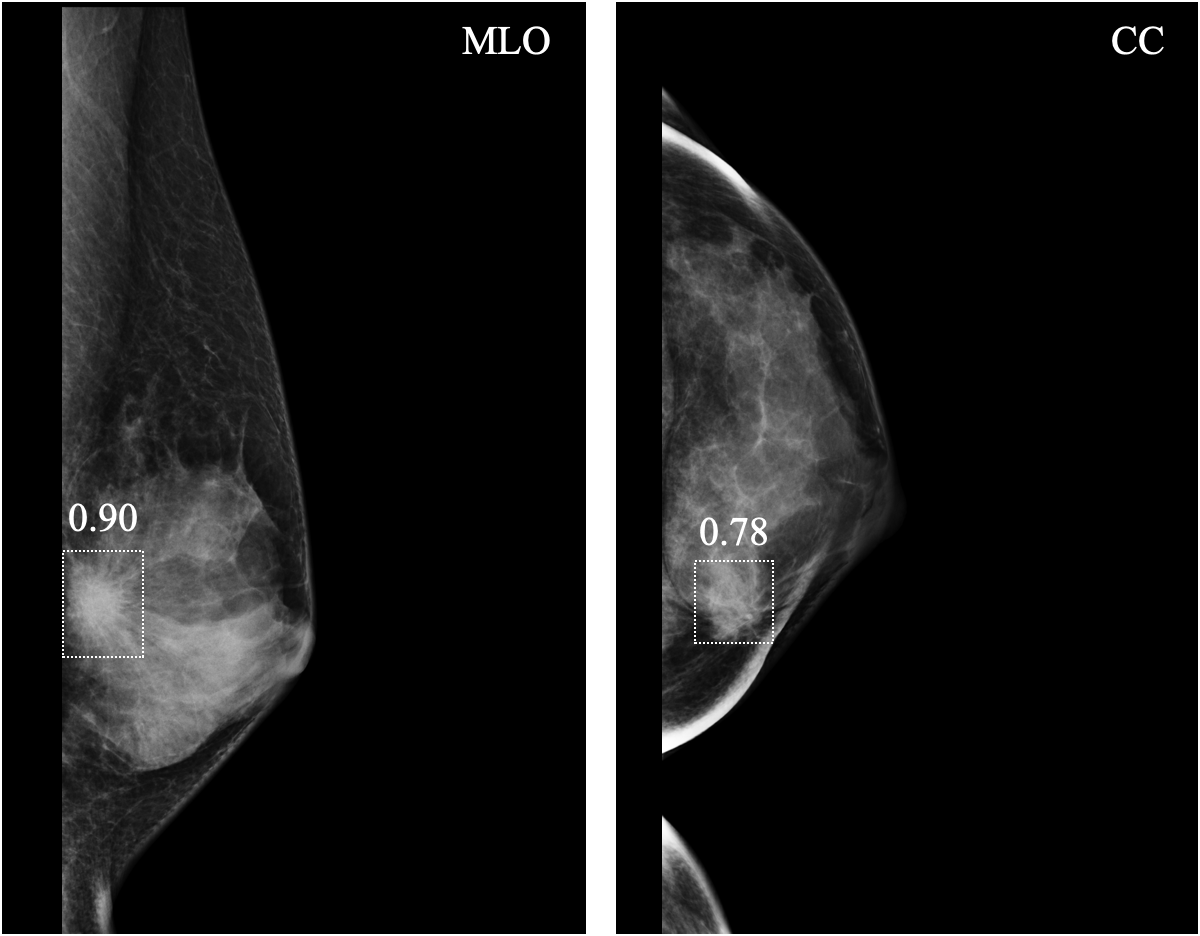


**Supplemental Figure 2 Free-response receiver operating characteristic (FROC) curves for our dataset using different DL models**


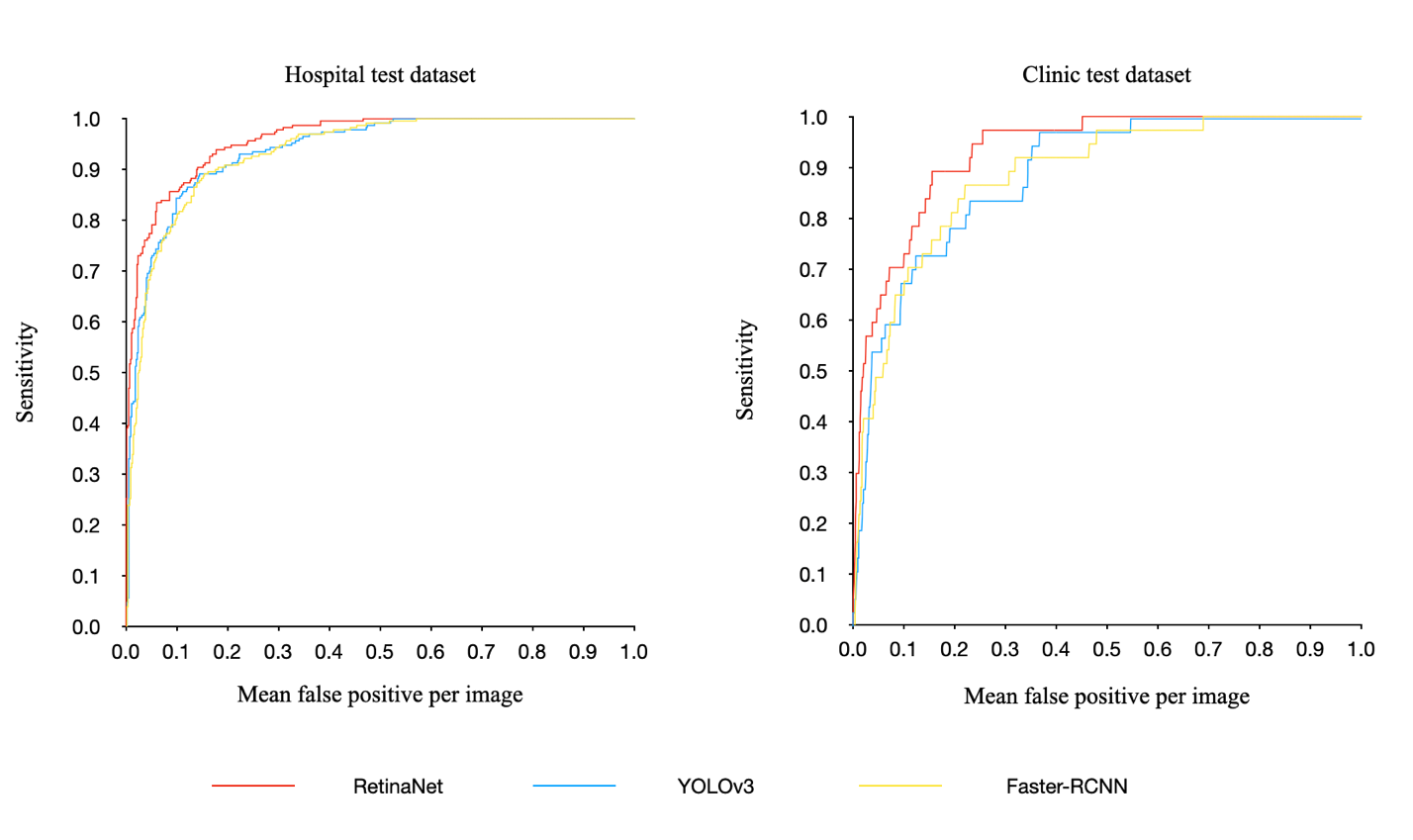


**Supplemental References**

1. Redmon J, Farhadi A. Yolov3: An incremental improvement. arXiv preprint arXiv:180402767. 2018.

2. Ren S, He K, Girshick R, Sun J. Faster R-CNN: Towards real-time object detection with region proposal networks. IEEE Trans Pattern Anal Mach Intell. 2017;39(6):1137-49. Epub 2016/06/14. doi: 10.1109/tpami.2016.2577031. PubMed PMID: 27295650.

3. Bunch PC, Hamilton JF, Sanderson GK, Simmons AH. Free response approach to measurement and characterization of radiographic observer performance. AJR Am J Roentgenol. 1978;130(2):382.
